# Application of a theoretical model to evaluate COPD disease management

**DOI:** 10.1186/1472-6963-10-81

**Published:** 2010-03-26

**Authors:** Karin MM Lemmens, Anna P Nieboer, Maureen PMH Rutten-Van Mölken, Constant P van Schayck, Javier D Asin, Jos AM Dirven, Robbert Huijsman

**Affiliations:** 1 Erasmus University Rotterdam, Institute of Health Policy and Management, P.O. Box 1738, 3000 DR Rotterdam, the Netherlands; 2 Erasmus University Rotterdam, Institute for Medical Technology Assessment (iMTA), Rotterdam, the Netherlands; 3 Department of General Practice, Maastricht University, Research Institute CAPHRI, Maastricht, the Netherlands; 4 ACSION, Advance Care Solutions and Insights for Optimization, Amstelveen, the Netherlands; 5 CAHAG, General Practitioners' Advisory Group for COPD and Asthma, Utrecht, the Netherlands

## Abstract

**Background:**

Disease management programmes are heterogeneous in nature and often lack a theoretical basis. An evaluation model has been developed in which theoretically driven inquiries link disease management interventions to outcomes. The aim of this study is to methodically evaluate the impact of a disease management programme for patients with chronic obstructive pulmonary disease (COPD) on process, intermediate and final outcomes of care in a general practice setting.

**Methods:**

A quasi-experimental research was performed with 12-months follow-up of 189 COPD patients in primary care in the Netherlands. The programme included patient education, protocolised assessment and treatment of COPD, structural follow-up and coordination by practice nurses at 3, 6 and 12 months. Data on intermediate outcomes (knowledge, psychosocial mediators, self-efficacy and behaviour) and final outcomes (dyspnoea, quality of life, measured by the CRQ and CCQ, and patient experiences) were obtained from questionnaires and electronic registries.

**Results:**

Implementation of the programme was associated with significant improvements in dyspnoea (p < 0.001) and patient experiences (p < 0.001). No significant improvement was found in mean quality of life scores. Improvements were found in several intermediate outcomes, including investment beliefs (p < 0.05), disease-specific knowledge (p < 0.01; p < 0.001) and medication compliance (p < 0.01). Overall, process improvement was established. The model showed associations between significantly improved intermediate outcomes and improvements in quality of life and dyspnoea.

**Conclusions:**

The application of a theory-driven model enhances the design and evaluation of disease management programmes aimed at improving health outcomes. This study supports the notion that a theoretical approach strengthens the evaluation designs of complex interventions. Moreover, it provides prudent evidence that the implementation of COPD disease management programmes can positively influence outcomes of care.

## Background

Due to aging of the population, numbers of persons suffering from chronic conditions are growing at astonishing rates. Chronic illnesses will be the primary cause of death and disability in the world by 2020 [1]. Among the most common chronic diseases is chronic obstructive pulmonary disease (COPD), which represents an enormous burden on individuals, families and societies, by its impact on quality of life, health resource utilisation, and mortality [2-4]. The causes and maintaining factors of chronic conditions are complex; therefore a multifaceted, multidisciplinary and multi-institutional response is needed [1]. Models of care coordination, such as disease management, capture the complexity of providing health care for chronic conditions in a coordinated manner. They also underscore the importance of using multifaceted approaches as opposed to "magic bullet" or single interventions [5]. Multifaceted programmes offer different combinations of interventions directed at patients, professionals and/or the organisation of care [6]. Although these programmes vary widely in structure and style, the primary goals - to improve disease outcomes while containing overall healthcare costs - tend to be consistent.

Yet it is the very heterogeneity of the programmes that makes it hard to evaluate them and compare them on results. An evaluation model has therefore been developed to structurally evaluate disease management programmes, in which theoretically driven inquiries link disease management interventions to outcomes achievement [6]. In this paper this model was applied to a COPD disease management programme implemented in the 'Gelderse Vallei' region in central Netherlands. Seven general practices to that aim cooperated with the regional hospital and home care organisation. The programme includes three main features: patient education, protocolised assessment and treatment of COPD, and coordination of care. This study reports on the effects of the implementation of this programme on process, intermediate and final outcome indicators as derived from the evaluation model. It is hypothesised that the implementation of multiple disease management interventions will influence intermediate and eventually final outcomes of care.

### Theoretical framework

**Figure 1:**
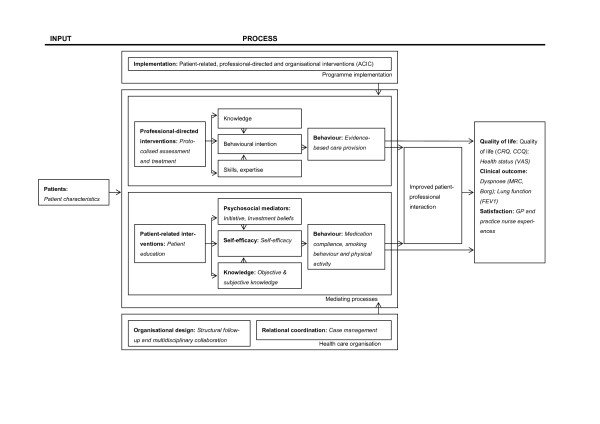
**Disease management evaluation model for the 'Gelderse Vallei' region**.

Determining the effectiveness of complex interventions requires understanding of the components of an intervention and their interrelationships ("black box") [6,7]. The theoretically derived evaluation framework, which is based on social learning theories, links the disease management components with the underlying mechanisms by which they influence outcomes, and proposes direct and indirect relationships among them. The framework has been described in detail elsewhere [6] and includes three key components: patient-related and professional-directed interventions, supported by the organisational design. Theoretical approaches on organisational and behavioural change are integrated, supporting the premise that combing these interventions strengthens the effects of disease management. The various components and indicators defined in this study are shown in Figure 1. A distinction was made between process indicators (what is done), intermediate indicators (procedural end point, i.e. behaviour change) and final outcome indicators (end results of care, i.e. change in patient health status) [8]. In this paper, the model will be put to the test for the very first time.

To test the model we will first examine the actual implementation of the COPD disease management programme in the 'Gelderse Vallei' region through process evaluation. Changes in intermediate and final outcomes of care are expected to result from programme implementation. Next, because both patient-related and professional-directed interventions aim to alter behaviour through mechanisms such as gaining knowledge, skills and/or self-efficacy we will also evaluate the extent to which evidence-based care is provided. And, finally, we will analyse if the presumed patient behavioural change - due to improving knowledge, psychosocial beliefs and self-efficacy - contributes to the attainment of expected outcomes.

## Methods

### Study design

The study was designed as an one-group pre-post test study [9]. This design was chosen to test the theoretical basis and components of this complex intervention and is also an opportunity to determine the consistency with which the intervention is delivered [10]. Composition of a control group was not feasible, due to involvement of other regional practices in the implementation of innovations which would bias the study of usual care practices.

### Participants and setting

The programme was carried out in seven general practices (12 general practitioners (GPs)) in the 'Gelderse Vallei' region in central Netherlands. The practices were solo (43%), duo (28%) and small group practices (28%) (on average 2700 patients per fulltime unit GP); most GPs were male (67%). Practice characteristics were representative of other practices in this region and also of Dutch practices in general (Additional file 1) [11,12]. These practices cooperated with the regional hospital. The practices were supported by practice nurses of a regional home care organisation. Patients were recruited between April 2006 and August 2006. All known COPD patients, as well as individuals who satisfied predefined criteria (aged ≥ 40, previous use of COPD or asthma medication and International Classification of Primary Care (ICPC) codes COPD (R95) or asthma (R96)), were invited for diagnostic assessment. The practice nurses identified eligible patients supported by the GP and an electronic protocol. Patients with a confirmed diagnosis of COPD, according to Global Initiative for Chronic Obstructive Lung Disease (GOLD) criteria which include confirmation by spirometry [13], were invited to participate, and informed consent was obtained. Patients with serious co-morbidity, for instance congestive heart failure, were not eligible for inclusion.

### Disease management interventions

The COPD disease management programme consisted of three facets (Additional file 2): One, a *patient-related intervention *designed to increase patients' understanding of the condition and to teach them specific prevention or treatment strategies on smoking behaviour, medication usage, nutrition and physical activity. In practice this intervention was offered during each contact for at least fifteen minutes (patient-education). An information booklet was provided as well. Secondly, a *professional-directed intervention *designed to educate professionals on the guidelines and programme (protocolised assessment and treatment of COPD). Over twelve months, GPs attended three courses on standardised COPD management and spirometry training. Before the introduction of the programme the practice nurses received six days of training in standardised COPD management and the provision of medical and non-medical treatment. The training was designed to reinforce knowledge on diagnosing COPD, assessing COPD severity, patient self-management, smoking cessation, follow-up, and planning possible action against exacerbations. Both GPs and practice nurses were trained in the application of spirometry. The implementation of the guidelines was supported by an electronic diagnostic and treatment protocol. Third, these interventions were supported by *organisational interventions *consisting of structural follow-up, case management and multidisciplinary collaboration. A multidisciplinary team cooperated in a system for coordinating diagnostic procedures, treatment, and ongoing patient management (coordination of care). The tasks and responsibilities of the members (GP, practice nurse and a lung specialist) were described in a guideline-based protocol. The practice nurses performed diagnostic tests such as lung function measurements, assessed patients' conditions, provided disease-related education and advice, coordinated care, and organised follow-up meetings at 3, 6 and 12 months. They acted in conjunction with the GP who consulted the specialist when needed. Generally, telespirometry was used to confirm the diagnosis; the test results in the general practice were dispatched through a simple telephone call to the lung specialist who was able to make a functional diagnosis.

### Data collection

Data on patient intermediate outcomes as well as quality of life and satisfaction were collected by means of postal questionnaires. The following patient characteristics were also gathered: age, gender, education and marital status. Professional behaviour and clinical outcome data were obtained from an electronic registry, which was part of the software protocol for treatment and monitoring of COPD patients in participating practices. Process data were gathered by means of a survey among professionals.

### Process

Actual exposure to the disease management programme was evaluated by the Assessing Chronic Illness Care (ACIC) survey [14]. In this study, only the process elements reflecting the programme's interventions were included: self-management support (patient-related intervention), decision support (professional-directed intervention), as well as delivery system design and clinical information system (both organisational interventions). Professionals rated the 4 to 6 items per element on a 1 to 11 scale, with higher scores indicating greater implementation. A mean score for each element was obtained. These data were validated with the care registries' process data

### Intermediate outcomes

Compliance and disease-specific knowledge were measured using a validated Dutch-language instrument [15]. Objective knowledge was measured by means of 22 true or false questions about COPD and expressed as the proportion of correct answers, transformed into a 0-10 scale. Subjective knowledge was assessed by means of six questions on a five-point scale indicating the estimation that patients make of their amount of knowledge on COPD. Self-reported medication compliance and physical activity practice were both measured by numeric rating (0-10) in combination with questions on a five-point scale that were expressed as one overall score. Next to that, self-reported smoking behaviour was measured on a dichotomous scale. Psycho-social mediators and self-efficacy were assessed using three dimensions of the Self-Management Ability Scale (SMAS): investment (the ability to invest in longer-term benefits) and initiative beliefs (the ability to be self-motivating regarding the realisation of the dimensions of well-being), as well as self-efficacy [16,17]. The subscales each consist of five items rated on a six-point scale. A higher score indicated better ability.

Professional behaviour was measured from the degree to which GPs applied evidence-based care after the intervention. Professional adherence to protocolised assessment and treatment of COPD was measured by four main guideline aspects: GOLD classification with each COPD diagnosis, application of spirometry, prescription of airway medication and inspection of patients' medication usage and technique. The application of patient education was evaluated from the proportions of patients reporting they had received clear information and information booklets. Continuity of care was assessed from the proportions of patients regularly followed. The indicators are expressed as percentages of patients that received a specific intervention.

### Final outcomes measures

A validated Dutch version of the self-reported Chronic Respiratory Questionnaire (CRQ-SR) was used to measure quality of life [18,19]. The CRQ-SR is made up of 20 questions and measures four dimensions relating to dyspnoea (5), emotional function (7), fatigue (4), and mastery (4). For every question there are seven response categories that score from 1 to 7; higher scores indicated better quality of life. A change of 0.5 in each dimension has been calculated as being the minimum clinically important difference (MCID) [19].

Additionally, the Clinical COPD Questionnaire (CCQ) was used, a self-administered multidimensional symptom control questionnaire that includes 10 questions in three domains: symptoms (4), functional (4) and mental state (2) [20]. The total CCQ score, and the score on each of the three domains, varies between 0 (very good health status) to 6 (extremely poor health status). An average change in score of 0.4 for the total score has been shown to be the MCID [20].

The EQ-5D visual analogue scale (VAS), a "thermometer" for eliciting a self-rating of health status, was used. Patients mark their perceived health status on a line with anchors 0 (worst imaginable health state) and 100 (best imaginable health state) [21].

The Medical Research Council (MRC) scale is a commonly used, validated, ordinal scale on which patients rate the type and magnitude of their dyspnoea according to five grades of increasing severity: 0 indicating 'breathlessness with strenuous exercise' to 5 representing 'breathlessness keeping patients from leaving the house' [22]. Dyspnoea was also measured by the modified Borg scale, a 0 to 10 rated scale to measure and evaluate patients' dyspnoea [23].

Lung function was assessed by measuring the forced expiratory volume in one second (FEV_1_) with the use of spirometry and expressed as FEV_1_percentage predicted, according to established criteria [13]. FEV_1 _was measured from a series of at least three forced expiratory curves that had an acceptable start of test and were free from artefact.

Patients' experiences on quality of care were measured using a self-administered questionnaire, the QUOTE (QUality Of care Through the patients' Eyes) COPD [24]. In this study, only indicators reflecting aspects of quality of care specifically targeted by the programme's interventions were included, namely coordination and accessibility of care, education on medication use and non-medical self-care.

### Statistical analyses

Comparisons for all study variables were made between baseline and 12 months post-intervention using paired-sampled *t*-tests and Wilcoxon signed rank tests (two-sided; α = 0.05) where appropriate. To determine if the theoretical model propositions are consistent with the data, regression analyses were applied in two steps. Intermediate indicators (independent variables) with significant changes in means entered the regression model with changes in outcomes as subsequent dependent variables. In accordance with the theoretical model first investment beliefs, subjective and objective knowledge entered the equation and then medication compliance. Even though outcome measures did not change significantly, within group variation in outcomes made it feasible to perform these regression analyses. Scores are arranged so that positive correlations indicate a positive relation. All analyses were performed at patient level. Data processing and analysis were performed using SPSS 15 for Windows. A prior significance level of 0.05 was used for all statistical tests.

## Results

### Patient inclusion, assignment and follow-up

Two hundred fifty-nine patients were found eligible to participate in the disease management programme, of whom 189 (73%) consented to participate in the study. Participants did not significantly differ at baseline with regard to sex, age and stage of COPD (p < 0.05) from the patients that did not participate in the study. Table 1 shows baseline characteristics of all participants and those for whom data collection was complete (150/189; 79%). Most patients had mild or moderate forms of COPD, which make up the majority of the COPD population [25]. A comparison of drop-outs with patients that completed the study revealed no clinical differences on any baseline characteristics and quality of life measures. The most common reason for dropping out of the study was unwillingness to complete questionnaires; questionnaires were resend when they were not returned.

**Table 1:** Baseline characteristics

*Baseline characteristics*	*Total population* *(n = 189)*	*Completed data* *(n = 150)*
	***Mean ± SD***	***Mean ± SD***

Age	66 (± 11)	66 (± 11)
Lung function (FEV_1_)	75.7 (± 18.6)	75.4 (± 18.6)
Tiffeneau	63.6 (± 11.4)	63.9 (± 11.1)
Dyspnoea	1.68 (± 1.09)	1.65 (± 1.07)
Body Mass Index	27.0 (± 4.2)	27.1 (± 4.3)

	***% (n)***	***% (n)***

Sex		
Male	65 (122)	67 (100)
Female	35 (67)	33 (50)
Marital status		
Married or equivalent	80 (149)	81 (120)
Widowed	10 (19)	11 (16)
Divorced	2 (3)	2 (3)
Never married	8 (15)	6 (9)
Educational level greater than high school diploma	24 (46)	26 (39)
Smoking status		
Non or Ex-smoker	74 (139)	77 (115)
Current smoker	26 (50)	23 (35)
COPD severity		
GOLD 1 (Mild)	47 (90)	46 (69)
GOLD 2 (Moderate)	40 (75)	43 (64)
GOLD 3 (Severe)	13 (24)	11 (17)

### Process evaluation

Table 2 summarises the extent to which the caregivers felt that the disease management programme was actually implemented. Although all scores increased, those for decision support and self-management support changed the least and were not significant. Scores on organisational interventions, delivery system design and clinical information system, had improved significantly after 12 months (p = 0.012 and p ≤ 0.001, respectively).

**Table 2:** Process improvement

***Dimension***^†^	*Baseline* *(Mean ± SD)*	*12 months* *(Mean ± SD)*	*Change* *(95% CI)*	*p-value*
Patient-related (Self-management support)	5.97 (± 2.44)	6.93 (± 2.11)	0.95 (-0.46; 2.36)	0.163
Professional-directed (Decision support)	6.88 (± 1.40)	7.86 (± 1.63)	0.98 (-0.13; 2.09)	0.079
Organisational (Delivery system design)	6.67 (± 1.45)	7.97 (± 1.51)	1.30 (0.35; 2.24)	0.012*
Organisational (Clinical information system)	5.83 (± 1.17)	7.03 (± 1.23)	1.20 (0.68; 1.73)	0.000*

^† ^Positive change means improvement

### Intermediate outcomes

Objective and subjective measurement of knowledge improved significantly (p = 0.002 and p ≤ 0.001, respectively). Variables related to self-efficacy and psychosocial beliefs remained the same or improved, the difference in investment beliefs being statistically significant (p = 0.049). Compliance with the medication regimen has improved after 12-months follow up (p = 0.003), unlike smoking status or physical activity practice (Table 3).

**Table 3:** Changes in intermediate outcomes

*Indicator*	*Baseline* *(Mean ± SD)*	*12 months* *(Mean ± SD)*	*Change* *(95% CI)*	*p-value*
***Psycho-social mediators***^†^				
Initiative beliefs (scale 1-6)	4.04 (± 0.74)	4.10 (± 0.77)	0.06 (-0.06; 0.18)	0.335
Investment beliefs (scale 1-6)	4.13 (± 0.76)	4.24 (± 0.74)	0.10 (0.01; 0.20)	*0.049*
***Self-efficacy***^†^				
Self-efficacy (scale 1-6)	4.24 (± 0.74)	4.23 (± 0.78)	-0.01 (-0.11; 0.10)	0.865
***Knowledge on COPD***^†^				
Subjective knowledge (scale 1-5)	2.84 (± 0.84)	3.22 (± 0.82)	0.38 (0.24; 0.51)	*0.000*
Objective knowledge (scale 1-10)	4.49 (± 2.10)	4.92 (± 2.03)	0.43 (0 .16; 0.69)	*0.002*
***Behaviour***				
Smoking status* (% smoking)	23%	22%	1%	0.180
Medication compliance^† ^(scale 1-5)	4.41 (± 0.63)	4.58 (± 0.58)	0.17 (0.05; 0.28)	*0.003*
Physical activity^† ^(scale 1-5)	2.80 (± 0.54)	2.85 (± 0.53)	0.05 (-0.02; 0.12)	0.167

* Negative change means improvement; ^† ^Positive change means improvement

With regard to professional behaviour, all patients were diagnosed with spirometry and were classified according to the GOLD criteria. Airway medication was mostly prescribed in line with guideline recommendations (92%). Correct use of medication by patients was checked for 92% of the patients. Seventy-eight percent of the patients reported to have received clear information (78%), however only 56% had received an information booklet. Ninety-two percent of patients regularly attended follow-up meetings.

### Final outcomes of care

Table 4 summarises the findings for all clinical outcomes, quality of life variables and patient experiences, comparing the baseline measurement with the 12-month measurement and determining statistical significance of the difference. Dyspnoea had improved significantly on both MRC (p ≤ 0.001) and Borg scale (p ≤ 0.001). A significant decline with regard to lung function was found. Clinically and statistically significant improvements were not found on quality of life scores (according to the CRQ, CCQ and VAS). Overall, patient experiences with practice nurses, as measured by QUOTE, had improved (p < 0.001). More specifically, improvements were found on the subscales accessibility, education on medication use and on non-medical self care. Improvement on patient experiences with GPs was only found for the subscale on accessibility (p = 0.016).

**Table 4:** Changes in outcomes of care

*Outcome indicator*	*Baseline* *(Mean ± SD)*	*12 months* *(Mean ± SD)*	*Change* *(95% CI)*	*p-value*
***Quality of life***				
CRQ^† ^(scale 1-7)				
CRQ (dyspnoea)	5.68 (± 1.33)	5.70 (± 1.33)	0.02 (-0.13; 0.16)	0.823
CRQ (emotional function)	5.39 (± 1.05)	5.34 (± 1.10)	-0.04 (-0.19; 0.10)	0.551
CRQ (fatigue)	4.79 (± 1.27)	4.85 (± 1.27)	0.53 (-0.10; 0.21)	0.515
CRQ (mastery)	5.63 (± 1.07)	5.64 (± 1.07)	0.01 (-0.17; 0.19)	0.895
CCQ (total)* (scale 0-6)	1.38 (± 0.84)	1.45 (± 0.92)	0.07 (-0.05; -0.19)	0.237
CCQ (symptoms)	1.86 (± 0.94)	1.83 (± 1.03)	-0.03 (-0.17; -0.11)	0.686
CCQ (functional)	1.34 (± 1.11)	1.47 (± 1.16)	0.13 (-0.02; 0.28)	0.087
CCQ (mental state)	0.54 (± 0.83)	0.66 (± 0.86)	0.11 (-0.02; 0.24)	0.088
Health Status (VAS)^† ^(scale 0-10)	6.99 (± 1.34)	6.97 (± 1.54)	-0.03 (-0.22; 0.16)	0.758
***Symptoms***				
Dyspnoea (MRC) (scale 0-5)*	1.61 (± 1.06)	1.30 (± 1.07)	-0.31 (-0.47; -0.15)	*< 0.001*
Dyspnoea (Borg) (scale 0-10)*	2.42 (± 1.62)	2.01 (± 1.28)	-0.41(-0.65; -0.17)	*0.001*
Lung function (FEV_1 _% predicted)^†^	76.93 (± 17.96)	73.59 (± 18.53)	-3.34 (-4.94; -1.75)	*< 0.001*
***Patient experiences***^†^				
General practitioner (scale 0-10)	6,95 (± 1.69)	7,00 (± 1.70)	0.46 (-0.32; 0.41)	0.804
Practice nurse (scale 0-10)	5,60 (± 1.87)	7,15 (± 1.44)	1.55 (0.82; 2.28)	*< 0.001*

* Negative change means improvement; ^† ^Positive change means improvement

### Testing the Evaluation Model's propositions

Table 5 shows the results of the hierarchical regression analyses that explored the associations between changes in intermediate outcomes and changes in outcomes of care. Changes in investment beliefs and/or subjective knowledge were found to be significant predictors of changes in CRQ, CCQ and health status scores. No direct or mediating effect was found of medication compliance on outcomes of care. Additional analyses showed that including all intermediate variables did not influence our results.

**Table 5:** Associations between significantly changed intermediate outcomes and changes in outcomes of care

Variables	CRQ Dyspnoea β		CRQ Mastery β		CCQ Total β		CCQ Mental state β		CCQ functional state β		Health Status (VAS) β		Borg β	
	**Step 1**	**Step 2**	**Step 1**	**Step 2**	**Step 1**	**Step 2**	**Step 1**	**Step 2**	**Step 1**	**Step 2**	**Step 1**	**Step 2**	**Step 1**	**Step 2**

1. Knowledge (O)	-0.05	-0.05	-0.04	-0.04	-0.01	-0.01	0.00	0.00	-0.04	-0.04	-0.10	-0.10	-0.19*	-0.19*
Knowledge (S)	0.12	0.13	0.24*	0.24*	0.15*	0.15*	0.18*	0.18*	0.30*	0.30*	0.29*	0.29*	0.30	0.31
Investment beliefs	0.26*	0.24*	0.30*	0.33*	-0.18	-0.17	0.05	0.03	0.27*	0.25*	0.15	0.14	0.21	0.18
2. Medication compliance		-0.14		0.16		-0.09		-0.07		-0.14		-0.06		-0.16
R^2^	0.06	0.08	0.08	0.09	0.07	0.08	0.04	0.05	0.13	0.14	0.08	0.08	0.07	0.08

*p < .05; **p < .01 (2-tailed) PAIRWISE

## Discussion

The objective of this study was to evaluate the effectiveness of a disease management programme for COPD patients according to the evaluation model and to explore associations between the model elements. Implementation of the programme was associated with significant improvements in dyspnoea and patient experiences with the practice nurses, whereas quality of life measures remained stable. It would seem, therefore, that symptoms may improve despite worsening of lung function parameters [26]. COPD is a progressive disease and lung function can be expected to worsen over time [27].

Improvements were also found in several intermediate outcomes, including investment beliefs, disease-specific knowledge and medication compliance. The model associations were examined, and investment beliefs and subjective knowledge proved to be predictors of quality of life and health status. This would indicate that increasing patients' ability to invest in longer-term benefits positively relates to outcomes of care. In contrast with our model, no direct or mediating effect was found of behaviour change. This may be due to the absence of significant changes in physical and smoking behaviour. However, direct relations between knowledge gain and investment beliefs (independent variables) and quality of life (dependent variable) were found; suggesting the direct influence of cognitive processes on quality of life.

The findings in our study are to some extent consistent with results from other studies on the effectiveness of COPD disease management: improved patient satisfaction and process measures [28] and inconclusive results on quality of life [29-32]. However, earlier studies focussed on single components [29] rather than on programmes that did include all three components. This study presents an example of a theory-based evaluation of disease management. This in contrast with studies on other complex interventions that are often defined pragmatically and lack any clear theoretical basis [33]. Process, intermediate as well as final outcome indicators should be selected on theoretical grounds, as was done in this study.

Several factors might have influenced the effect size of the programme in this study. The multidimensional and multidisciplinary nature of disease management in addition to the amount of time it takes to detect changes, presents further challenges in developing an evidence base within a time and financially limited research project. The 12 months follow-up period may have been too brief to observe all changes resulting from the intervention, since major effects of disease management interventions are expected to occur in the long term [34]. Over time, changes found in intermediate outcomes may be predictors of improvements, particularly in quality of life. Moreover, the patients in this study had less severe disease than those in some other studies, potentially reducing possibilities to detect improvement in our sample. And finally, even though all process implementation measures improved, full implementation of the programme was not always reached. For example, evidence-based care provision by professionals was hindered by distribution problems of the information booklet to the practice nurses.

Furthermore, intensity of the patient-related intervention, which is crucial for improving skills, ability to cope with illness, and health status [13], may have been too low. This could explain why changes in self-efficacy were not observed. And even though physical activity is an important predictor of outcomes in COPD care [35], it did not change significantly as a result of the intervention. Greater attention to physical activity and smoking cessation is indicated, therefore. The inclusion of a more comprehensive self-management component within the programme, such as motivational interviewing instead of patient education, seems most promising to this end [36].

Findings of this study must be interpreted in the light of several limitations. First, the lack of a control group means that it can not be assumed that positive effects of this study were solely due to the programme [37]. However, we found no reason to assume that elements such as the introduction of new medications on the health care market or changes in the insurance system occurred that could have caused improvements of comparable magnitude. The included general practices were representative for other Dutch practices. Since randomisation is not feasible in such a setting a viable alternative design would be interrupted time series or a delayed treatment design [38]. Yet, a theory-driven approach, as used in this study, to understanding complex social interventions and their effects is very valuable. Nevertheless we fully acknowledge that results have to be interpreted with caution. Second, self-reported instruments were used. Self-reporting of medication and exercise behaviour must be interpreted as an estimate of particular behaviours. Still, the multi-item scales used met standards for reliability, and support for validity has been reported for several measures [15,16,19,20]. Third, data on cost-effectiveness were not gathered, due to separate registration systems within general practices and the hospital. Considering that ever tightening budgets cannot meet the continuously increasing demand for healthcare, it is important to assess the costs and cost effectiveness of disease-management programmes. And finally, not all proposed model associations have been tested, because of the absence of significant changes in some intermediate outcomes. Previous research already showed associations between behaviour and quality of life in cross-sectional data [39]. Future research should therefore focus on testing associations between changes in patient and professional behaviour (independent variables) and changes in outcomes of care (dependent variables).

## Conclusions

The application of a theoretical model improves the design and evaluation of disease management programmes. It helps to understand the context and the processes of the intervention, and to select the appropriate indicators for evaluation. Although results have to be interpreted with caution due to the research design, this study provides prudent evidence that the implementation of disease management for patients with COPD can positively influence outcomes of care. Moreover, stronger effects may emerge in the long run.

## Competing interests

The research project was supported by an unrestricted grant from PICASSO for COPD, an initiative of Pfizer BV and Boehringer Ingelheim BV in cooperation with research institute Caphri (Care and Public Health Research Institute) of Maastricht University. The author(s) declare that they have no competing interests.

## Authors' contributions

KMML designed and carried out the evaluation study, performed the statistical analysis, and drafted the manuscript. APN and RH designed the study and have been involved in drafting the manuscript. MPHMR, CPS and JDA participated in the design of the study and read drafted components and contributed to refinement of the manuscript. JAMD supported the acquisition of data and contributed to refinement of the manuscript. All authors have read and approved the final manuscript.

## Pre-publication history

The pre-publication history for this paper can be accessed here:

http://www.biomedcentral.com/1472-6963/10/81/prepub

## Supplementary Material

Additional file 1 **Practice characteristics**. An overview of practice characteristics of the included general practices in this study as compared to characteristics of other general practices in the Netherlands. Click here for file

Additional file 2 **Disease management interventions in the 'Gelderse Vallei' region**. An overview of the content of the interventions which were implemented as a part of the disease management programmes Click here for file
