# Supplementary material for: Application of a theoretical model to evaluate COPD disease management
Source: BMC Health Serv Res. 2010 Mar 26;10:81. doi: 10.1186/1472-6963-10-81 (PMC2859741; doi:10.1186/1472-6963-10-81)
Supplement: Additional file 1 — Practice characteristics. An overview of practice characteristics of the included general practices in this study as compared to characteristics of other general practices in the Netherlands. [file 1472-6963-10-81-S1.DOC]

| **Characteristics** |  | **Lemmens et al.** | | **Van Avendonk et al. 2007#** | **Schellevis et al. 2004‡** | **Meulepas et al. 2008^** | |
| --- | --- | --- | --- | --- | --- | --- | --- |
|  |  | *Project* | *Project region* |  |  | *Intervention* | *Control* |
| Number of GPs |  | 12 | 102 | 1621 | 8209 |  |  |
| Number of practices |  | 7 | 50 |  |  | 23 | 28 |
| GP characteristics | male | 67% | 62% | 76% | 69% | – | – |
|  | practice experience | 20 ± 7 year | 19 ± 8 year | 18 ± 9 year* | – | – | – |
|  |  |  |  |  |  |  |  |
| Practice type | single | 43% | 40% | 37% | 37% | 39% | 42% |
|  | duo | 28% | 38% | 33% | 32% | – | – |
|  | group | 28% | 22% | 31% | 31% | – | – |
|  |  |  |  |  |  |  |  |
| Average population size/FTE general practitioner |  | 2700 ± 412* | 2606 ± 490* | 2705 ± 614* | 2392 | 2729 ± 267* | 2741 ± 379* |
|  |  |  |  |  |  |
| Practice organisation | Physician assistant available | 100% | – | 55% | – | 100% | 100% |

* = Mean ± SD

# Van Avendonk MJP, Gorter KJ, van den Donk M, Rutten GEHM. **Niet alle huisartsen hebben de praktijkorganisatie om optimale diabeteszorg te leveren. Een vragenlijstonderzoek.** *Huisarts Wet* 2007;**50**(11):529-34

**‡** Schellevis FG, Westert GP, de Bakker DH, Groenewegen PP: **Tweede Nationale Studie naar ziekten en verrichtingen in de huisartspraktijk. Vraagstellingen en methoden.** Utrecht / Bilthoven: NIVEL / RIVM 2004

^ Meulepas MA, Braspenning JC, de Grauw WJ, Lucas AE, Wijkel D, Grol RP: **Patient-oriented intervention in addition to centrally organised checkups improves diabetic patient outcome in primary care.** *Qual Saf Health Care* 2008, 17(5):324-328
