# Supplementary material for: Application of a theoretical model to evaluate COPD disease management
Source: BMC Health Serv Res. 2010 Mar 26;10:81. doi: 10.1186/1472-6963-10-81 (PMC2859741; doi:10.1186/1472-6963-10-81)
Supplement: Additional file 2 — Disease management interventions in the 'Gelderse Vallei' region. An overview of the content of the interventions which were implemented as a part of the disease management programmes [file 1472-6963-10-81-S2.DOC]

| **Patient-related intervention:** *Patient education* |
| --- |
| - More emphasis on systematic use of patient education, including the following topics: smoking behaviour, medication usage, nutrition and physical activity   - Frequency: every 3 months   - Duration: First contact ten minutes, follow-up meetings at least fifteen minutes   - Provision of information booklet |
| **Professional-directed intervention:** *Protocolised assessment and treatment of COPD* |
| - Educational meetings GPs and practice nurses on the following topics: COPD management according to guidelines and application of spirometry. Practice nurses also concentrated on the provision of patient education: smoking behaviour, medication usage, nutrition and physical activity   - Frequency: GP - three times a year; practice nurse – 6 days at the start   - Duration: GP – two hours; practice nurse eight hours (each session) - Active distribution of clinical practice guidelines   - An electronic decision support system assists GPs and practice nurses in diagnosis, therapy and treatment of patients - Audit and feedback by lung specialist/ peers   - Every spirometry test to confirm COPD diagnosis was seen by the lung specialist for confirmation, additionally other spirometry tests could be send to the lung specialist when necessary   - GPs received feedback data from their practice after the start of the programme and after 12 months |
| **Organisational intervention:** *Coordination of care* |
| - Arrangements for structural follow-up - Periodical follow-up meetings at 3, 6 and 12 months with practice nurse. Multidisciplinary collaboration   - Multidisciplinary care teams, each composed of a GP, practice nurse and a lung specialist   - Revision of professional roles; more delegation of care from GP to practice nurse, and from lung specialist to GP. The practice nurses discussed their findings with GPs - Case management   - Care coordination by the practice nurse in collaboration with the GP |
